# Non-alcoholic Wernicke encephalopathy following pancreaticoduodenectomy (Whipple procedure): a case report and review of the literature

**DOI:** 10.1097/RC9.0000000000000989

**Published:** 2026-09-16

**Authors:** Ricardo Mitsuru Hanada-Angulo, Frank Young-Tabusso

**Affiliations:** a Department of Internal Medicine, Clínica Oncosalud-Auna, Lima, Peru; b Department of Hepatopancreatobiliary Surgery, Clínica Oncosalud-Auna, Lima, Peru

**Keywords:** case report, non-alcoholic, pancreaticoduodenectomy, SIADH, thiamine deficiency, Wernicke encephalopathy

## Abstract

**Introduction and importance:**

Wernicke encephalopathy (WE) is an acute neurological emergency caused by thiamine deficiency. It is uncommon after pancreaticoduodenectomy and may be overlooked when postoperative metabolic disturbances coexist.

**Presentation of case:**

A 66-year-old woman underwent pancreaticoduodenectomy for a duodenal neoplasm. Her postoperative course was complicated by pancreatic fistula, extended-spectrum beta-lactamase-producing *Escherichia coli* infection, impaired oral intake, and prolonged nutritional support. During the third to fourth postoperative weeks, she developed fluctuating encephalopathy, bradylalia, and horizontal nystagmus. Serum thiamine was 1.2 µg/dL (reference range: 2.8–8.5 µg/dL), and magnetic resonance imaging showed periaqueductal T2/fluid-attenuated inversion recovery hyperintensity. Hypoosmolar hyponatremia with clinical euvolemia and preserved thyroid and adrenal function was considered compatible with probable postoperative syndrome of inappropriate antidiuretic hormone secretion, although urinary biochemical confirmation was unavailable. Intravenous thiamine 500 mg every 8 hours for 3 days, followed by 250 mg daily for 5 days, produced partial improvement within 24–72 hours. At 3-month follow-up, the neurological manifestations had resolved.

**Clinical discussion:**

Pancreaticoduodenectomy and its complications can accelerate thiamine depletion within a broader spectrum of postoperative nutritional and metabolic disturbances. The oculomotor findings, low serum thiamine, characteristic imaging findings, and treatment response supported a diagnosis of WE. Hyponatremia probably contributed to the altered mental status but did not explain the ocular signs or periaqueductal lesions.

**Conclusion:**

WE should be considered in postoperative patients with nutritional compromise and new neurological or oculomotor findings. Parenteral thiamine should not be delayed while awaiting confirmation, and coexisting electrolyte disorders should be corrected cautiously.

## Introduction

Wernicke encephalopathy (WE) is an acute neurological syndrome caused by thiamine deficiency. Although classically associated with alcohol use disorder, it also occurs in non-alcoholic patients with malignancy, prolonged fasting, impaired gastrointestinal absorption, critical illness, or incomplete nutritional supplementation. The classical triad of altered mental status, ocular motor dysfunction, and gait ataxia is often incomplete, which contributes to delayed diagnosis^[^1^]^.

HIGHLIGHTS

- Wernicke encephalopathy may follow a complicated pancreaticoduodenectomy.
- Oculomotor signs should prompt immediate intravenous thiamine.
- Periaqueductal magnetic resonance imaging changes support, but do not define, the diagnosis.
- Postoperative hyponatremia may coexist with and obscure the presentation.

Patients undergoing major gastrointestinal surgery are particularly vulnerable because thiamine stores are limited and postoperative complications may involve reduced intake, malabsorption, infection, and increased metabolic demand. Cancer-related gastrointestinal surgery has been associated with clinically relevant thiamine deficiency^[^2^]^. Only a small number of WE cases have been described after pancreaticoduodenectomy, including presentations during a complicated early postoperative course and several years after surgery^[^3–7^]^.

This report describes non-alcoholic WE after pancreaticoduodenectomy, complicated by pancreatic fistula and infection. The simultaneous presence of hypoosmolar hyponatremia, considered compatible with postoperative syndrome of inappropriate antidiuretic hormone secretion (SIADH), created an additional diagnostic challenge.

## Methods

This case report has been reported in line with the SCARE 2025 checklist^[^8^]^.

### Case presentation

#### Patient information and postoperative course

A 66-year-old woman with no relevant documented medical or surgical history and no reported history of chronic alcohol use was admitted to a private tertiary-care hospital for a neoplasm of the second portion of the duodenum. She underwent pancreaticoduodenectomy (Whipple procedure).

The early postoperative course was characterized by conjugated hyperbilirubinemia and hypoalbuminemia. Markedly elevated drain amylase concentrations on postoperative days 2 and 3 raised suspicion of a postoperative pancreatic fistula. Culture of fluid from the lower abdominal drain grew extended-spectrum beta-lactamase-producing *Escherichia coli*. The pancreatic fistula and associated intra-abdominal infection were managed conservatively with systemic antimicrobial therapy (piperacillin–tazobactam followed by ertapenem), nutritional support, and serial surveillance of the intra-abdominal collections, without the need for surgical, endoscopic, or radiological intervention. The highest postoperative complication severity was therefore classified as Clavien–Dindo grade II^[^9^]^. The chronological sequence is summarized in Table 1.

**Table 1:** Timeline of the clinical course.

Time point	Event details
Postoperative day 0	Pancreaticoduodenectomy (Whipple procedure) was performed for a second-portion duodenal neoplasm.
Postoperative days 2 and 3	Markedly elevated drain amylase raised suspicion of a postoperative pancreatic fistula.
Postoperative day 6	Drain culture identified ESBL-producing *Escherichia coli*; broad-spectrum antimicrobial therapy was continued.
Postoperative days 11–16	Intra-abdominal collections were monitored, and nutritional support was maintained; no surgical, endoscopic, or radiological reintervention was required.
Postoperative days 20–23	Serum sodium ranged from 124 to 129 mmol/L while the patient remained clinically stable.
Postoperative day 26	Serum osmolality was 265 mOsm/kg; thyroid and adrenal function were preserved, and probable postoperative SIADH was suspected, although urine osmolality and urine sodium were unavailable.
Postoperative day 29	Fluctuating mental status, bradylalia, and horizontal nystagmus were documented. MRI showed periaqueductal T2/FLAIR hyperintensity; serum thiamine was 1.2 µg/dL.
Postoperative days 29 and 30	High-dose intravenous thiamine was initiated.
Within 24–72 hours	Bradylalia and oculomotor abnormalities partially improved.
3-month follow-up	Horizontal nystagmus and the remaining neurological manifestations had resolved.

ESBL, extended-spectrum beta-lactamase; FLAIR, fluid-attenuated inversion recovery; MRI, magnetic resonance imaging; SIADH, syndrome of inappropriate antidiuretic hormone secretion.

#### Clinical findings and diagnostic assessment

During the third postoperative week, the patient developed fluctuating mental status, slowed speech, and horizontal nystagmus. She had a Glasgow Coma Scale score of 15, remained oriented, and had no documented focal neurological deficit. Gait ataxia was not reliably documented during the postoperative period.

Serum sodium fluctuated between 124 and 135 mmol/L, with a measured serum osmolality of 265 mOsm/kg. Clinical euvolemia, a thyroid-stimulating hormone concentration of 3.05 mIU/L, and a morning cortisol concentration of 26.7 µg/dL supported a presumptive diagnosis of postoperative SIADH. However, urine osmolality and urine sodium were not obtained during the hyponatremic episode; therefore, the complete biochemical diagnostic criteria for SIADH could not be fulfilled, and SIADH was considered a probable clinical diagnosis rather than a biochemically confirmed diagnosis. Serum thiamine was markedly reduced at 1.2 µg/dL (reference range: 2.8–8.5 µg/dL).

Brain magnetic resonance imaging (MRI) showed periaqueductal hyperintensity on T2-weighted and fluid-attenuated inversion recovery sequences, with chronic leukoaraiosis and no acute ischemic or hemorrhagic lesion (Fig. 1). The differential diagnosis included hyponatremic encephalopathy, medication-related neurotoxicity, sepsis-associated encephalopathy, and an acute structural or vascular lesion. The combination of nutritional risk, horizontal nystagmus, low thiamine concentration, characteristic MRI findings, and subsequent response to thiamine strongly supported WE.

**Figure 1.**
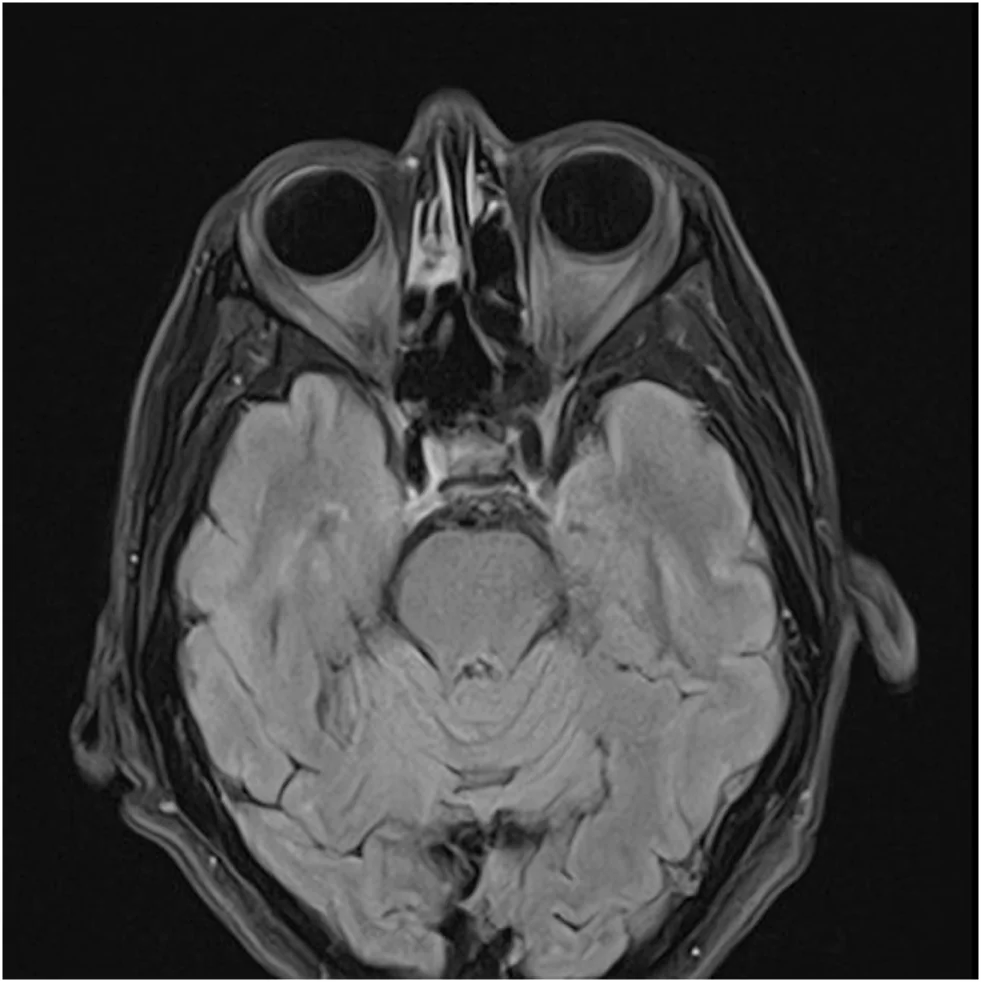
Axial fluid-attenuated inversion recovery brain MRI demonstrating periaqueductal hyperintensity, supportive of Wernicke encephalopathy.

#### Therapeutic intervention and follow-up

High-dose parenteral thiamine was started immediately: 500 mg intravenously every 8 hours for 3 days, followed by 250 mg intravenously once daily for an additional 5 days. The coexisting hypoosmolar hyponatremia was managed with fluid restriction and serial serum sodium monitoring; controlled hypertonic saline was reserved for neurologic deterioration or severe symptomatic hyponatremia.

Bradylalia and oculomotor abnormalities improved partially within 24–72 hours after initiation of thiamine. At outpatient follow-up 3 months later, horizontal nystagmus had resolved, and the patient had recovered from the remaining neurological manifestations.

## Discussion

This case illustrates WE developing within weeks of a complicated pancreaticoduodenectomy. The postoperative pancreatic fistula, intra-abdominal infection, reduced intake, hypoalbuminemia, and prolonged nutritional support probably acted together to accelerate thiamine depletion. The rapid onset is biologically plausible because body thiamine reserves are small and may be exhausted within a few weeks when intake is inadequate and metabolic demand is increased^[^1^]^.

Published experience after pancreaticoduodenectomy remains limited. Onieva-Gonzalez *et al* described WE after cephalic pancreaticoduodenectomy and prolonged parenteral nutrition without thiamine supplementation^[^3^]^. Karayiannakis *et al* reported WE after pancreaticoduodenectomy for pancreatic cancer^[^4^]^. Kim *et al* described confusion, horizontal nystagmus, and ataxia after a complicated pylorus-preserving pancreaticoduodenectomy with pancreatic fistula and prolonged poor oral intake^[^5^]^. Monden *et al* reported WE after pancreaticoduodenectomy with pancreatic fistula and metabolic complications^[^6^]^, whereas AbdelRazek *et al* documented a delayed presentation 5 years after a Whipple procedure^[^7^]^. The present case adds an early postoperative presentation after surgery for a duodenal neoplasm and highlights the confounding effect of concomitant hypoosmolar hyponatremia.

Pancreaticoduodenectomy may also produce nutritional and metabolic consequences beyond an isolated thiamine deficiency. Altered gastrointestinal anatomy, reduced pancreatic exocrine function, maldigestion, impaired oral intake, and postoperative catabolic stress may contribute to pancreatic exocrine insufficiency, protein–calorie malnutrition, and multiple micronutrient deficiencies; endocrine dysfunction, including new-onset diabetes, is also a recognized long-term consequence. Recent evidence supports systematic attention to exocrine function, micronutrient status, and glycemic health after pancreatic resection^[^10–12^]^. In the present patient, thiamine deficiency should therefore be viewed as one manifestation within a broader postoperative nutritional and metabolic vulnerability.

The diagnosis of WE remains primarily clinical. MRI is supportive but insufficiently sensitive to exclude the disease; a classic study reported a sensitivity of 53% and a specificity of 93%^[^13^]^. Typical lesions involve the medial thalami, mammillary bodies, periaqueductal gray matter, and regions adjacent to the third and fourth ventricles^[^13,14^]^. In this patient, periaqueductal hyperintensity, horizontal nystagmus, a markedly reduced serum thiamine concentration, and improvement after parenteral replacement formed a coherent diagnostic pattern. Treatment should not be deferred until imaging or laboratory confirmation because neurological injury may become irreversible^[^1^]^.

Hypoosmolar hyponatremia may independently cause lethargy, cognitive fluctuation, seizures, or encephalopathy. It was, therefore, a plausible contributor to the altered mental status. Nevertheless, probable postoperative SIADH did not account for the oculomotor abnormalities or the characteristic periaqueductal MRI changes. In patients at increased risk of osmotic demyelination, including those with malnutrition or hypokalemia, serum sodium correction should be conservative and closely monitored^[^15^]^.

The principal strengths of this report are the convergence of clinical, biochemical, radiological, and therapeutic-response data and the documented 3-month neurological outcome. Limitations include the absence of urine osmolality and urine sodium, which precluded complete biochemical confirmation of SIADH; accordingly, SIADH remained a probable clinical diagnosis. Additional limitations include the lack of a reliably documented gait examination during the acute postoperative period and the absence of a formal patient-perspective statement. As a single case, the report cannot establish incidence or causality. Nonetheless, it supports comprehensive postoperative nutritional surveillance and a low threshold for empiric parenteral thiamine when compatible neurological findings arise after pancreaticoduodenectomy.

## Conclusion

Non-alcoholic WE is an uncommon but preventable neurological emergency after pancreaticoduodenectomy. Complicated postoperative recovery with poor intake, infection, and prolonged nutritional support should prompt active consideration of thiamine deficiency within a broader nutritional and metabolic assessment. New-onset encephalopathy with oculomotor findings warrants immediate parenteral thiamine, even when concurrent metabolic abnormalities, such as hyponatremia, could partially explain the presentation.

## Data Availability

Data sharing is not applicable to this article, as no datasets were generated or analyzed for this case report. Relevant de-identified clinical information is contained within the article.

## References

[1] Sechi GP Serra A. Wernicke’s encephalopathy: new clinical settings and recent advances in diagnosis and management. Lancet Neurol 2007;6:442–55. 10.1016/S1474-4422(07)70104-7 17434099

[2] Restivo A Carta MG Farci AMG. Risk of thiamine deficiency and Wernicke’s encephalopathy after gastrointestinal surgery for cancer. Support Care Cancer 2016;24:77–82. 10.1007/s00520-015-2748-z 25931232

[3] Onieva-Gonzalez FG Blanco-fernandez G Munuera-romero L. Wernicke’s encephalopathy after cephalic pancreaticoduodenectomy. Rev Esp Enferm Dig 2011;103:594–96. 10.4321/s1130-01082011001100009 22149564

[4] Karayiannakis AJ Souftas V Bolanaki H. Wernicke encephalopathy after pancreaticoduodenectomy for pancreatic cancer. Pancreas 2011;40:1157–59. 10.1097/MPA.0b013e31822182f5 21926559

[5] Kim JS Rho SY Hwang HK. A case of Wernicke’s encephalopathy following complicated laparoscopic pylorus-preserving pancreaticoduodenectomy. Ann Hepatobiliary Pancreat Surg 2019;23:295–99. 10.14701/ahbps.2019.23.3.295 PMC6728253 31501822

[6] Monden K Sadamori H Hioki M. Wernicke’s encephalopathy after pancreaticoduodenectomy: a case report. Case Rep Clin Nutr 2020;2:1–7.

[7] AbdelRazek M Han C Albrecht A. Wernicke’s encephalopathy 5 years after a Whipple procedure. Neurohospitalist 2018;8:NP3–NP4. 10.1177/1941874417710878 PMC5882004 29623164

[8] SCARE Group, Kerwan A Al-jabir A Mathew G. Revised Surgical CAse REport (SCARE) guideline: an update for the age of artificial intelligence. Prem J Sci 2025;10:100079.

[9] Dindo D Demartines N Clavien PA. Classification of surgical complications: a new proposal with evaluation in a cohort of 6336 patients and results of a survey. Ann Surg 2004;240:205–13. 10.1097/01.sla.0000133083.54934.ae PMC1360123 15273542

[10] Phillips ME Livingstone C Frampton AE. Micronutrient deficiencies after pancreatico-duodenectomy: a narrative review of the literature and recommendations for clinical practice. Nutr Clin Pract 2025;40:1073–92. 10.1002/ncp.70007 PMC12450337 40736420

[11] Di Martino M de la Hoz Rodriguez A Saibanti A. Pancreatic exocrine insufficiency after pancreatic resection: a systematic review. BMC Surg 2025;25:53. 10.1186/s12893-025-02787-y PMC11789297 39901161

[12] Beger HG Poch B Mayer B. New onset of diabetes and pancreatic exocrine insufficiency after pancreaticoduodenectomy for benign and malignant tumors: a systematic review and meta-analysis of long-term results. Ann Surg 2018;267:259–70. 10.1097/SLA.0000000000002422 28834847

[13] Antunez E Estruch R Cardenal C. Usefulness of CT and MR imaging in the diagnosis of acute Wernicke’s encephalopathy. AJR Am J Roentgenol 1998;171:1131–37. 10.2214/ajr.171.4.9763009 9763009

[14] Zuccoli G Pipitone N. Neuroimaging findings in acute Wernicke’s encephalopathy: review of the literature. AJR Am J Roentgenol 2009;192:501–08. 10.2214/AJR.07.3959 19155417

[15] Spasovski G. Hyponatraemia-treatment standard 2024. Nephrol Dial Transplant 2024;39:1583–92. 10.1093/ndt/gfae162 39009016

